# Homocysteine and education but not lipoprotein (a) predict estimated 10-year risk of cardiovascular disease in blood donors: a community based cross-sectional study

**DOI:** 10.1186/s12872-019-1157-5

**Published:** 2019-07-26

**Authors:** Francesco Vadini, Francesca Santilli, Giuseppe Casalini, Mario dell’Isola, Ornella Iuliani, Damiano D’Ardes, Luisa Lattanzio, Marta Di Nicola, Giancarlo Di Iorio, Patrizia Accorsi

**Affiliations:** 1 FIDAS (Italian Federation of Associations of Blood Donors), Pescara, Italy; 2 Psychoinfectivology Service, Infectious Disease Unit, Pescara General Hospital, Pescara, Italy; 3 Department of Medicine and Aging, “G. d’Annunzio” University of Chieti-Pescara, Chieti, Italy; 4 Department of Hematology, Transfusion Medicine and Biotechnologies, Pescara General Hospital, Pescara, Italy; 5 Clinical Pathology Unit, Pescara General Hospital, Pescara, Italy; 6 Center of Aging Science and Translational Medicine (CESI-Met), “G. D’Annunzio” University Foundation, Via Luigi Polacchi, 66013 Chieti, Italy

**Keywords:** Blood donors, Cardiovascular risk, Homocysteine, Education

## Abstract

**Background:**

With aging of the population, screening and prevention health programs for blood donors will increasingly be a priority. We aimed at: assessing the 10 year-cardiovascular disease (CVD) risk in blood donors, according to Italian CUORE risk score (CRS); determining the association of homocysteine (Hcy), lipoprotein (Lp)(a) and socio-demographic or lifestyle variables with estimated 10-year CVD risk.

**Methods:**

Between June 2015 and July 2017, 1,447 (61.2% men) unselected blood donors (aged 18–69 years) were enrolled at the Blood Transfusion Service of the Pescara General Hospital, Italy. The project entailed evaluation of unalterable (age and gender) and modifiable CV risk factors (total cholesterol, HDL, LDL, triglycerides, fasting glucose, smoking, hypertension). The educational attainment, socio-demographic and lifestyle behavior information were obtained through a structured self-report questionnaire, and Health-related quality of life (HRQoL) through the Short Form Survey (SF-12). Plasma Hcy and Lp(a) were determined in the fasting state.

**Results:**

A CRS within the moderate-high risk range was reported in 21.7% donors. Multivariate logistic regression, after adjustment for clinical and demographic variables, showed that Hcy [OR (95% CI): 1.09 (1.04–1.13); *p* < 0.001) and low educational attainment [1.71 (1.09–2.73); *p* = 0.019] are independent risk factors for moderate-to-high CVD risk. Instead, Lp(a), evaluated in 774 donors, was > 30 mg/dL in 22.4% of the examined population, but without any significant correlation with CRS.

**Conclusions:**

Our study highlights a previously unappreciated need for CV risk assessment in blood donors, which may include evaluation of educational attainment as a non-traditional risk marker.

## Background

Risk assessment and prevention represent an important goal to provide safe and effective blood products and services to the community and to promote excellent transfusion medicine practices and research. The overarching goals of blood center–based health screening programs are to improve the community’s health, to preserve donor eligibility and to incentivize individuals to donate. Screening and prevention health programs for the population of blood donors will increasingly be a priority, because the population structure in most countries is shifting from younger to older age groups because of an increase in life expectancy and aging of previous high birth rate cohorts, aggravated by a sustained decrease in birth rates. Because most blood donors belong to the age group of 18 (or 16 years in some countries) to 65 years and the majority of blood recipients are in the age group > 65 years, the ratio between these 2 population groups is relevant for the blood supply/demand relationship. This ratio will change substantially during the next decade [1]. In this context, cardiovascular disease (CVD) risk prevention is a priority. CVD is the primary cause of premature death in developed nations [2]. Angina, myocardial infarction, and other clinical outcomes of CVD result from atherosclerosis, a progressive inflammatory condition that contributes to “clinically silent” or preclinical changes in arterial morphology. Traditional risk factors for developing CVD include obesity, hypertension, dyslipidemia, diabetes mellitus, smoking, age and sex. Several scientific societies recommend screening some biomarkers of cardiometabolic risk, along with traditional risk factors [3–8]. In this study we evaluated only two biomarkers, homocysteine (Hcy) and Lp(a), although no consensual recommendations exist for routine testing. Aims of this study were: (1) to evaluate traditional and non traditional cardiovascular risk factors, including homocysteinemia and lipoprotein (a); (2) to assess the 10 year-risk for CVD in blood donors over the age of 35 years, according to Italian CUORE risk score (CRS); (3) to determine the association of Hcy and Lp(a) with estimated 10-year risk of CVD; (4) to determine the association of sociodemographic and lifestyle variables with estimated 10-year risk of CVD.

## Methods

### Population and study design

A representative sample of unselected blood donors was included in this cross-sectional study. Among data available for blood donors aged between 18 and 70 years, referring to the Blood Transfusion Service of the Pescara General Hospital, Italy between June 2015 and July 2017, cardiometabolic risk factors, biochemical indicators such as homocysteine and Lp (a), and lifestyle behaviors were analyzed.

#### Data collection

### Socio-demographic, education and lifestyle behavior

Data were collected as part of the usual clinical practice. Written informed consent was obtained from each blood donor included in the study, the study protocol conforms to the ethical guidelines of the 1975 Declaration of Helsinki.

Socio-demographic characteristics, schooling, and lifestyle behavior information were obtained through a structured self-report questionnaire with the constant guidance of an operator. We recorded the number of cigarettes smoked per day. Subjects smoking at least one cigarette a day were considered as smokers. Physical activity was defined as sedentary lifestyle (no regular activity < 1 time/week), and regular physical activity (≥ 1 time/week). We asked participants for the years of education plus possible training course. The years of education has been investigated for its implications with life style, disease and aging, according to relevant studies correlating education and ageing-related diseases such as dementia and CVD [9–12]. Blood donors with years of education below the 25th percentile (corresponding to less than 13 years of education) were considered as having low educational attainment (LEA), those with at least 13 years of education were categorized as high educational attainment (HEA).

### Anthropometric and health data measurements

Height (cm), weight (kg) and waist circumference (cm) were measured by trained occupational health nurses during the occupational health examination. The waist circumference was measured in the horizontal plane of the superior border of the iliac crest with the individual in standing position after a full expiration. Body mass index (BMI) was calculated as weight (kilograms) divided by the square of height (metres). Resting blood pressure (BP) was measured after 5 minutes in a seated position with an automated BP monitor using the oscillometric method. Any systolic BP found to be140 mmHg and/or diastolic BP 90 mmHg was verified with a standard mercury sphygmomanometer after a two-minute interval. Arterial blood pressure was measured in one arm. At least two blood pressure measurements were taken, in a sitting position,1–2 min apart, and additional measurements if the first two were quite different. The average value of the different measurements was considered.

### Laboratory analysis

Biochemical tests were centralized at the Laboratory of Clinical Chemistry of Pescara General Hospital. The following parameters were assessed using standard analytical techniques: fasting blood glucose (FBG), total cholesterol (TC), high-density lipoprotein cholesterol (HDL-C) and triglycerides (TG). Fasting plasma Hcy was measured by HemosIL™ Homocysteine assay, an automated, latex-enhanced immunoassay for the quantitative determination of total plasma homocysteine (Hcy) (Instrumental Laboratory, Bedford, MA, USA). Lp(a) plasma concentrations were determined by IMMAGE® LPA immunonephelometric assay (IMMAGE LPA), performed on the IMMAGE® Immunochemistry System (Beckman Instrument, Inc., Galway, Ireland).

### Ten-year cardiovascular risk estimate

The 10-year cardiovascular risk was assessed using the CRS built within the Italian CUORE project. The CUORE project (epidemiology and prevention of ischemic heart diseases) is a prospective, fixed-cohort study, including cohorts from the north, the center, and the south of Italy, coordinated by the Italian NIH (National Institute of Health). The CUORE project risk score allows estimating the probability of experiencing a first cardiovascular event over the next 10 years, based on the level of eight cardiovascular risk factors: age, gender, total blood cholesterol (TC), high-density lipoprotein-cholesterol (HDL-C), systolic blood pressure (SBP), diabetes mellitus, smoking habit, and use of antihypertensive medication. The risk score is validated in patients 35 to 69 years of age without previous major cardiovascular events, followed-up over time in longitudinal studies [13, 14]. The CRS showed similar discrimination power compared with Framingham and European SCORE equations, but is more appropriate for the Italian population avoiding overestimation of the risk in a population with a low incidence of cardiovascular disease [13, 15]. On the basis of the CRS, patients are classified as low risk (CRS < 3.0%), risk to be kept under control through the adoption of a healthy lifestyle (CRS 3.0–19.9%), and high risk (CRS ≥ 20.0%).Since high CV risk is uncommon in blood donors, our population was divided into two groups: low risk (< 3%) and moderate-to-high risk (≥3%). Data were collected using the software cuore.exe, downloadable free of charge from the website of the CUORE project http://www.cuore.iss.it/.

### Health-related quality of life (HRQoL)

Patients self-reported their perceived HRQoL using the widely used Short Form Survey (SF-12). The SF-12 is a well-known generic self-report health status instrument that includes a subset of 12 items from SF-36 [16]. Information from all 12 items is used to calculate a physical component score (PCS) representing the physical health domain and a mental component score (MCS) representing the mental health domain.

### Statistical analysis

The quantitative variables were summarized as mean and standard deviation (SD). The qualitative variables were summarized as frequency and percentage. Pearson’s Chi Square test was assessed to evaluate differences in demographic and clinical parameters between low and moderate/high risk groups of CRS and between Hcy and Lp (a) levels groups. Student T test was performed to evaluate differences of continuous variables between low and moderate/high CVD risk and Hcy and Lp (a) levels groups. Univariate logistic regression analyses were performed to estimate the risk of moderate/high CRS. Risk was synthetized as crude odds ratios (ORs) with 95% confidence intervals (95% CI). Multiple logistic regression models were performed to estimate the risk of moderate/high CRS for every 1 umol/L increase in Hcy and in LEA group, adjusted for clinical and demographic variables. Relative adjusted OR with 95% CI were computed. Alpha error was evaluated at 0.05. Statistical analysis was performed using the Stata 2013 version 13.1 software (Stata Corp., College Station, Texas, USA).

## Results

A total of 1,447 blood donors (61.2% men; aged between 18 and 69 years) were enrolled in this study after informed consent obtained from all participants. General characteristics, distribution of CVD risk factors and clinical parameters of the overall study population are reported in Table 1, as well as the characteristics of the two blood donors subgroups stratified according tolow and moderate to high 10-years CVD risk. The CRS was calculated in 1,129 participants. Two-hundred and twenty-one (15.3%) blood donors were excluded from the calculation of CVD risk because the CRS is not applicable for subjects under 35 years. Of the 1,129blood donors, 884 (78.3%) were at low cardiovascular risk, while 245 (21.7%) donors reported a score within the moderate-high risk range. No participant was classified as high risk (CRS ≥ 20.0%). Higher CVD risk was associated with male gender (*p* < 0.001), smoke (*p* < 0.001), higher BMI (*p* < 0.001), waist circumference (*p* < 0.001), and LEA (*p* = 0.021) (Table 1). Moreover fasting glucose (*p* < 0.001), systolic and diastolic pressure (*p* < 0.001), total and LDL cholesterol (*p* < 0.001) and triglycerides (*p* < 0.001) were higher, whereas HDL cholesterol was lower (*p* < 0.001)in subjects with higher CVD risk (Table 1).

**Table 1:** Clinical, biochemical, behavioral and socio-demographic characteristics of blood donors stratified according to estimated 10-year cardiovascular disease risk

	Overall	Low CRS N (%) = 884 (78.3)	Moderate/High CRS N (%) = 245 (21.7)	*p-value*
Age, n (%) [*N* = 1,447]				*< 0.001*
18-29^a^	120 (8.3)	**–**	**–**	
30–40	298 (20.6)	183 (21)	1 (0.4)	
41–50	638 (44.1)	525 (59.4)	47 (19.2)	
51–60	322 (22.3)	160 (18.1)	146 (59.6)	
> 60	67 (4.6)	16 (1.8)	51 (20.4)	
Gender, n(%)				*< 0.001*
Male	886 (61.2)	462 (52.3)	225 (92)	
Female	561 (38.8)	422 (47.7)	20 (8.2)	
Marital Status, n (%) [*N* = 1,420]				*0.001*
Unmarried	331 (23.3)	131 (15)	17 (7)	
Married	972 (68.4)	663 (75.9)	195 (81.2)	
Divorced/separated	105 (7.4)	74 (8.5)	22 (9.2)	
Widow/er	12 (0.85)	5 (0.6)	6 (2.5)	
Educational attainment, n (%) [1,385]				*0.007*
HEA	1.098 (79.3)	671 (78.8)	167 (70.5)	
LEA	287 (20.7)	181 (21.2)	70 (29.5)	
Smokers, n (%) [*N* = 1,436]				*< 0.001*
Yes	299 (20.8)	135 (15.3)	72 (29.5)	
No	1.137 (79.2)	746 (84.7)	172 (70.5)	
Cigarettes/day, mean (SD)	1.9 (4.9)	1.4 (4.2)	3 (5.8)	*< 0.001*
Regular physical activity, n (%) [*N* = 1,436]				*0.177*
Yes	299 (20.8)	509 (57.7)	129 (52.9)	
No	1.137 (79.2)	373 (42.3)	115 (47.1)	
Physical activity/week, mean (SD)	1.6 (1.7)	1.6 (1.7)	1.3 (1.5)	*0.038*
Anthropometric indices, mean (SD)				
BMI (Kg/m^2^), [*N* = 1,435]	25.8 (3.9)	25.6 (3.7)	27.1 (3.5)	*< 0.001*
Waist circumference (cm), [*N* = 1,242]	91.3 (11.8)	90.3 (10.8)	97 (10.7)	*< 0.001*
CVD RF measurements, mean (SD)				
Fasting Glucose (mmol/l), [*N* = 1,415]	5.40 (12.5)	5.37 (12.4)	5.74 (12.7)	*< 0.001*
Systolic Blood Pressure (mm/Hg), [1,396]	120.4 (7.2)	119.4 (6.1)	125.6 (8.7)	*< 0.001*
Diastolic Blood Pressure (mm/Hg)	75.6 (6.2)	75 (5.9)	79.5 (5.7)	*< 0.001*
Total Cholesterol (mmol/l), [1,424]	5.11 (35)	5.11 (33.7)	5.56 (35.5)	*< 0.001*
Triglycerides (mmol/l)	1.16 (75)	1.07 (58.9)	1.63 (100.1)	*< 0.001*
HDL Cholesterol (mmol/l), [*N* = 1,360]	1.42 (13.3)	1.46 (13.4)	1.25 (11)	*< 0.001*
LDL Cholesterol (mmol/l), [*N* = 1,284]	3.58 (36.2)	3.56 (34.4)	4.07 (35.2)	*< 0.001*
Biomarkers, mean (SD)				
HCY (μmol/l), [*N* = 1,038]	11 (4.4)	10.7 (4.3)	12.5 (4.7)	*< 0.001*
Lp (a) (mg/dl), [*N* = 774]	20.3 (24.4)	21.5 (24.6)	19.4 (25.8)	*0.345*
Biomarkers prevalence, n (%)				
HCY ≥ 15 μmol/l	118 (11.4)	65 (9.7)	39 (18.7)	*< 0.001*
Lp (a) > 30 mg/dl	172 (22.4)	116 (24.2)	35 (20.3)	*0.308*
Health-Related Quality of life, mean (SD), [1,189]				
PCS	52.6 (5.6)	52.4 (5.7)	52.1 (5.8)	*0.575*
MCS	48.6 (9.4)	48.6 (9.4)	49.2 (8.8)	*0.398*

^a^Cuore Risk Score (CRS) is applicable to people with at least 35 years of age

*HEA* High educational attainment, *LEA* Low educational attainment, *BMI* Body Mass Index; *CVD RF* Cardiovascular Disease Risk Factors, *HDL* High Density Lipoprotein, *LDL* Low Density Lipoprotein, *Hcy* Homocysteine, *Lp(a)* Lipoprotein (a); *PCS* Physical Health Component Summary, *MCS* Mental Health Component Summary

### Hcy, Lp(a), CVD traditional risk factors and 10-year CV risk

The distribution of CVD risk factors in blood donors groups with high vs. low Hcy and Lp (a) levels is shown in Table 2. High levels of homocysteine (> 15 μmol/l) were observed in 11.4% of the total sample, and were associated with male gender (*p* < 0.001), higher BMI (*p* = 0.004), higher waist circumference (*p* = 0.005), lower HDL levels (*p* = 0.022), higher fasting plasma glucose levels (*p* = 0.010) and higher values of systolic blood pressure (*p* = 0.003). In fact, as shown in Table 1, the percentage of donors with high Hcy is higher among those with a CRS ≥3% (9.7 vs. 18.7%; *p* < 0.001). Instead, Lp(a) levels, evaluated in 774 donors, were higher than 30 mg/dL in 22.4% of the examined population, but they did not show any statistically significant correlation with CVD risk according to CRS (Table 1). With regards to lipid profile, Lp(a) has been shown to correlate directly with higher levels of total cholesterol (*p* = 0.002) and LDL cholesterol (*p* = 0.002) (Table 2). The multivariate logistic regression models (Table 3), after adjustment for clinical and demographic variables, confirmed that Hcy represents an independent risk factor for moderate-to-high CVD risk in our cohort of blood donors (OR: 1.09; 95% CI: 1.04 to 1.13; *p* < 0.001).

**Table 2:** Clinical, biochemical and socio-demographic characteristics of blood donors stratified according to homocysteine and lipoprotein (a) levels

	Overall	Hcy < 15 N (%) = 920 (88.6)	Hcy ≥15 N (%) = 118 (11.4)	*p-value*	Lp (a) < 30 N (%) = 597 (77.6)	Lp (a) ≥30 N (%) = 172 (22.4)	*p-value*
Age, n (%) [*N* = 1,445]				*0.253*			*0.560*
18–29	120 (8.3)	67 (7.3)	3 (2.5)		44 (7.4)	7 (4.1)	
30–40	299 (20.6)	182 (19.8)	23 (19.5)		96 (16.1)	26 (15.1)	
41–50	638 (44.1)	389 (42.4)	51 (42.5)		256 (42.9)	75 (43.6)	
51–60	322 (22.3)	237 (25.7)	33 (27.9)		168 (28.1)	55 (32)	
> 60	68 (4.6)	44 (4.8)	9 (7.6)		33 (5.5)	9 (5.2)	
Gender, n(%)				*< 0.001*			*0.011*
Male	886 (61.2)	542 (59.1)	103 (87.3)		396 (66.3)	96 (55.8)	
Female	561 (38.8)	375 (40.9)	15 (12.7)		201 (33.7)	76 (44.2)	
Marital Status, n (%) [*N* = 1,420]				*0.631*			*0.840*
Unmarried	331 (23.3)	195 (21.4)	20 (17.1)		117 (19.8)	34 (20)	
Married	972 (68.4)	633 (69.6)	87 (74.4)		420 (71.1)	120 (70.6)	
Divorced/separated	105 (7.4)	71 (7.8)	8 (6.8)		46 (7.8)	15 (8.8)	
Widow/er	12 (0.8)	10 (1.1)	2 (1.7)		8 (1.3)	1 (0.6)	
Educational attainment, n (%) [*N* = 1,286]				*0.820*			*0.859*
HEA	1,016 (79)	715 (79.4)	91 (78.4)		453 (77.4)	129 (76.8)	
LEA	270 (21)	186 (20.6)	25 (21.5)		132 (22.6)	39 (23.2)	
Smokers, n (%) [*N* = 1,436]				*0.576*			*0.701*
Yes	299 (20.8)	189 (20.7)	27 (22.9)		123 (20.6)	33 (19.3)	
No	1,137 (79.2)	726 (79.3)	91 (77.1)		473 (79.4)	138 (80.7)	
Cigarettes/day, mean (SD)	2 (4.9)	1.9 (4.9)	2.4 (5.4)	*0.375*	2 (4.8)	2 (5.7)	*0.843*
Regular physical activity, n (%) [*N* = 1,437]				*0.869*			
Yes	823 (57.3)	520 (56.8)	68 (57.6)		335 (56.2)	96 (56.1)	*0.987*
No	614 (42.7)	395 (43.2)	50 (42.4)		261 (43.8)	75 (43.9)	
Physical activity/week, mean (SD)	1.6 (1.7)	1.5 (1.6)	1.5 (1.8)	*0.877*	1.4 (1.5)	1.6 (1.9)	*0.154*
Anthropometric indices, mean (SD)							
BMI (Kg/m^2^), [*N* = 1,435]	25.8 (3.9)	25.7 (3.7)	26.8 (4.3)	*0.004*	26 (3.7)	25.8 (3.8)	*0.569*
Waist circumference (cm), [*N* = 1,242]	91.3 (11.8)	90.8 (11.8)	94.1 (12.2)	*0.005*	91.5 (12.3)	90.7 (11.6)	*0.499*
CVD RF measurements, mean (SD)							
Fasting Glucose (mmol/l), [*N* = 1,415]	5.40 (12.5)	5.41 (11.5)	5.58 (13.6)	*0.010*	5.46 (10.6)	5.45 (17.3)	*0.870*
Systolic Blood Pressure (mm/Hg), [1,396]	120.5 (7.2)	120.2 (6.7)	122.2 (7.8)	*0.003*	120.9 (7.8)	119.8 (5.9)	*0.083*
Diastolic Blood Pressure (mm/Hg)	75.6 (6.2)	75.4 (6.1)	76.4 (6.9)	*0.100*	75.8 (6.5)	75.3 (6.0)	*0.398*
Total Cholesterol (mmol/l), [1,424]	5.11 (35)	5.11 (35.2)	5.12 (33.6)	*0.942*	5.08 (33.1)	5.33 (35.8)	*0.002*
Triglycerides (mmol/l)	1.16 (75)	1.13 (67.9)	1.23 (62.7)	*0.193*	1.17 (73)	1.09 (57.4)	*0.251*
HDL Cholesterol (mmol/l), [*N* = 1,360]	1.42 (13.3)	1.40 (12.9)	1.32 (12.3)	*0.022*	1.38 (12.6)	1.43 (12.3)	*0.087*
LDL Cholesterol (mmol/l), [*N* = 1,344]	3.58 (36.2)	3.63 (36.6)	3.70 (35.7)	*0.468*	3.61 (37.3)	3.87 (36.3)	*0.002*
Quality of life, mean (SD), [1,189]							
SF-12 PCS	52.6 (5.6)	52.5 (5.7)	52 (5.9)	*0.377*	52.6 (5.3)	52 (6.2)	*0.181*
SF-12 MCS	48.6 (9.4)	48.4 (9.3)	49.4 (9.3)	*0.320*	48.6 (8.9)	47.7 (10.7)	*0.266*

**Table 3:** Increased risk (95% Confidence Intervals) for moderate/high 10-Year Cardiovascular Disease according to Italian CUORE Risk Score (CRS), estimate by logistic regression models

	CRS 10-Year CVD risk			CRS 10-Year CVD risk		
	OR (95% CI) Homocysteine (μmol/l)	*P*	R^2^	OR (95% CI) Low educational	*P*	R^2^
Model 1	1.08 (1.049–1.121)	< 0.001	0.025	1.55 (1.124–2.148)	0.008	0.006
Model 2	1.08 (1.049–1.123)	< 0.001	0.041	1.52 (1.095–2.110)	0.012	0.014
Model 3	1.08 (1.050–1.124)	< 0.001	0.053	1.49 (1.067–2.082)	0.019	0.028
Model 4	1.09 (1.047–1.126)	< 0.001	0.112	1.59 (1.114–2.296)	0.011	0.098
Model 5	1.09 (1.048–1.134)	< 0.001	0.179	1.69 (1.164–2.472)	0.006	0.153
Model 6	1.09 (1.046–1.138)	< 0.001	0.263	1.61 (1.070–2.428)	0.022	0.237
Model 7	–	–	–	1.71 (1.092–2.703)	0.019	0.263

Model 1 = Unadjusted; Model 2 = Model 1 and further adjusted for socio-demographic variable (marital status and educational attainment); Model 3 = Additionally adjusted for lifestyle (cigarettes/day and physical activity/week); Model 4 = Additionally adjusted for anthropometric variable (BMI and waist circumference); Model 5 = Additionally adjusted for diastolic blood pressure; Model 6 = Additionally adjusted for metabolic risk factors (LDL-Cholesterol, triglycerides and fasting glucose). Model 7 = Additionally adjusted for homocysteine

### Educational attainment, CVD traditional risk factors and 10-year CV risk

Table 1 shows that blood donors with LEA are more prevalent in the subgroup with moderate-to-high CVD risk as compared to low CVD risk subgroup (29.5 vs. 21.2%, *p* = 0.007). Consistently, the percentage of blood donors with HEA is higher in the low-CVD risk subgroup (78.8 vs. 70.5%). Blood donors with LEA were characterized more frequently by a sedentary lifestyle (54% vs. 39% in those with HEA (*p* < 0.001). In fact, blood donors with LEA compared to those with HEA had higher BMI (26.3 vs. 25.7; *p* = 0.032), higher waist circumference (92.7 vs. 91; *p* = 0.031), systolic blood pressure (121.3 vs. 120.2; *p* = 0.027), diastolic blood pressure (76.3 vs. 75.4; *p* = 0.029), total cholesterol (5.23 vs. 5.07; *p* = 0.009), and greater fasting plasma glucose levels (5.50 vs.5.36; *p* = 0.003). The multivariate logistic regression models (Table 3), after adjustment for clinical, biochemical and sociodemographic variables, confirmed that LEA represents an independent risk factor for moderate-to-high CVD risk in our cohort of blood donors (OR: 1.71; 95% CI: 1.09 to 2.73; *p* = 0.019).

## Discussion

Blood donors are generally referred to as healthy individuals at low CV risk. In fact they are usually considered as subjects in “good health”, generally without infectious diseases, and the presence of CVD is itself a contraindication to donation [17].

In this study, CV risk was estimated using the CRS, since the Framingham risk score seems to overestimate risk when applied to Mediterranean populations, such as our blood donors group [14, 15, 18, 19]. Conversely, the CRS has been shown to exhibit similar discrimination power compared with other predictive equations, by reducing risk overestimation [13, 18, 20]. The CUORE predictive equation has been previously used for blood donors and populations with a low incidence of CVD [13, 21, 22].

Interestingly, more than one fifth of blood donors were at moderate/high CV risk, a prevalence consistent with a large Italian study conducted on over 11,000 blood donors [22]. In addition, a non-negligible part of the subjects scoring a CV risk profile were aged under 50 years. These lines of evidence suggest that, unlike the common sense, blood donors are worth of screening for CV risk and, if eligible, potential candidates for primary prevention strategies. Thus, the use of a risk score may represent an important instrument to detect blood donors with considerable CV risk. The information derived from their CV risk profile may serve to increase awareness of health-related risks in both the blood donors and their general practitioners, and may be instrumental for actively changing lifestyle habits or for introducing therapy, if necessary. It should be emphasized that our blood donors were more frequently male (61% vs 38%), and male and female donors show a significantly different CV risk profile, despite comparable age (45.6 vs. 44.8 y, respectively). In our study, 35% of male donors vs. only 4% of female donors reported an estimated 10-year risk greater than 3%. These data are comparable with the results of the CARDIORISK study conducted on a large Italian population of blood donors, where 32% of male donors had reported a 10-year risk greater than 3% against 5.7% of women. Along these lines, a recent large prospective study in blood donors documented that the protective effect of donation frequency on the incidence of cardiovascular diseases was restricted to women and not observed in men [23]. This observation suggests that male sex represents a major cardiovascular risk factor even in blood donors, who would also gain less benefit from frequent donations.

In addition to chart risk score, biomarkers may have a valuable role. In particular Hcy has been shown to correlate with CVD and with coronary artery disease and myocardial infarction in observational and mendelian randomization studies [24, 25].

In our study, the levels of Hcy track with CV risk, with higher levels in blood donors at moderate/high CV risk profile. In fact, the prevalence of Hcy levels above the threshold is two-fold higher in subjects at moderate/high CV risk vs. those at low risk (18.7% vs. 9.7%). Thus, Hcy may represent a useful biomarker to identify CV risk in this peculiar population.

High levels of Lp(a), an LDL particle linked to the plasminogen-like glycoprotein, represent an independent risk factor for atherosclerotic CV disease, particularly in patients with high LDL-C or non-HDL-C [26–28]. Consistent with this, 22.4% of our population exhibited high levels of Lp(a). Surprisingly, and unlike homocysteine, Lp(a) has not shown any relationship with CV risk. The reason for this apparent discrepancy is unclear. Previous studies have reported that regular blood donation is associated with the lowering of parameters of lipid profile [29]. This finding has been explained in light of the evidence that regular blood donation reduces iron stores and this in turn may lower lipid peroxidation, and eventually cardiovascular risk [23]. We can speculate that Lp(a) levels may also be affected by reduced iron stores, thus disrupting the habitual relationship between Lp(a) and cardiovascular risk observed in the general population. This hypothesis should be confirmed by further studies.

Whatever the reason for such dissociation, lack of correlation between Lp (a) and CV risk score in the population of blood donors, if confirmed in larger cohorts, strengthens the importance to evaluate this non-traditional risk factor, with the potential to detect a portion of the population not highlighted by risk scores, exploring only classical risk factors.

In 2015, a Scientific Statement from the American Heart Association highlighted the importance of social determinants of risk and outcomes for cardiovascular disease [30]. The premise underlying this scientific statement is that, at present, the most significant opportunities for reducing death and disability from CVD lie with addressing the social determinants of cardiovascular outcomes. Among social factors, education provides the most consistent results in relation to CVD outcomes. The level of education is the main social factor used as a proxy for cognitive reserve [9–11, 31]. Cognitive Reserve was postulated to explain individual differences in susceptibility to ageing, offering apparent protection to those with higher education [32, 33]. In previous studies, adjustment for biological, CV and behavioral risk factors attenuated the effect of education on CVD incidence by up to 70% [12, 34–38]. These and other studies pointed to smoking, BMI, hypertension, lipid profile, and diabetes as mediators of the association between education and CVD incidence [35–37, 39–44], in agreement with our results.

We believe that a major strength of this study is the evaluation of CV risk biomarkers such as Hcy, Lp(a), rarely investigated in a population considered “healthy”. This study represents a first line of evidence on the importance of evaluating Hcy and “social factors” for the prevention of cardiovascular diseases, in an attempt to protect the health of the blood donor and eventually of blood supply. However, a number of limitations have to be pointed-out: given the cross-sectional nature of the study, a larger sample would have led to more solid conclusions. Furthermore, the donation frequency for each donor was not evaluated in this study. According to the iron-heart hypothesis, confirmed by recent longitudinal studies [23], high-frequency blood donation may exert a protective effect against cardiovascular disease.

## Conclusion

Our study highlights a previously unappreciated need for CV risk screening in the setting of blood donors, which may include assessment of the educational attainment as an additional marker of risk. Future studies are needed to demonstrate the usefulness of biomarkers in this setting. Meanwhile, CV risk assessment and CV primary prevention campaigns should be pursued with the aim of reducing mortality and morbidity and the associated social and human costs in this large population whose CV risk has been for long underestimated.

## Data Availability

The datasets used and/or analyzed during the current study are available from the corresponding author on reasonable request.

## Abbreviations

BMI: Body mass index
CRS: CUORE risk score
CVD: Cardiovascular disease
Hcy: Homocysteine
HRQoL: Health-related quality of life
Lp(a): Lipoprotein (a)
MCS: Mental component score
OR: Odds ratio
PCS: Physical component score
SF-12: Short form Survey-12
